# The prognostic value of fibrinogen to albumin ratio in malignant tumor patients: A meta-analysis

**DOI:** 10.3389/fonc.2022.985377

**Published:** 2022-09-29

**Authors:** Baibei Li, Huachu Deng, Biao Lei, Leijie Chen, Xinyuan Zhang, Dingran Sha

**Affiliations:** ^1^ Department of Hepatobiliary Surgery, The First Affiliated Hospital of Guangxi Medical University, Nanning, China; ^2^ Department of Gastrointestinal Surgery, the First Affiliated Hospital of Guangxi Medical University, Nanning, China; ^3^ The First Affiliated Hospital of Guangxi Medical University, Nanning, China; ^4^ Department of Urology Surgery, The First Affiliated Hospital of Guangxi Medical University, Nanning, China

**Keywords:** fibrinogen to albumin ratio, prognosis, meta-analysis, malignant tumor, biomarker

## Abstract

**Background:**

Recent studies have shown that the fibrinogen to albumin ratio (FAR) is closely related to the prognosis of various cancers. The aim of this systematic review and meta-analysis was to investigate the prognostic value of FAR in malignancies based on the available evidence.

**Method:**

To systematically search the Cochrane Library, Embase, PubMed, Google Scholar, Baidu scholars, CNKI and VIP databases for relevant studies published before April 1, 2022, and to evaluate the fibrinogen-to-albumin ratio (FAR) and survival of patients with malignant tumors through a meta-analysis relationship between the results. Results. This meta-analysis included 19 eligible studies involving 5926 cancer patients. We found that high FAR was associated with poor overall survival (HR=2.25, 95%CI 1.86-2.74, p<0.001), recurrence-free survival (HR=2.29, 95%CI 1.91-2.76, P<0.001), progression-free survival (HR: 2.10, 95%CI 1.58-2.79, p<0.001), disease-free survival (HR=1.52, 95%CI 1.17-1.96, p=0.001), and time to recurrence (HR: 1.555, 95%CI 1.031-2.346, P=0.035) was significantly correlated.

**Conclusions:**

High FAR is significantly associated with poor clinical outcomes in cancer, suggesting that it may be an important predictor of prognosis in patients with malignancies.

## Introduction

According to the latest data released by the American Cancer Society, 19.3 million new cancer cases and approximately 10 million deaths from cancer are expected worldwide in 2022 (1). As one of the leading causes of human death worldwide, how to prevent and treat cancer is a major issue in increasing the life expectancy of all human beings (2). Therefore, it is very important to find simple, effective and cheap markers to predict the prognosis of tumor patients. In recent years, many studies have shown that some biomarkers reflecting inflammation, nutritional status and immunity are related to the prognosis of cancer, including lymphocyte to monocyte ratio (LMR), neutrophil-to-lymphocyte ratio (NLR), fibrinogen to pre-albumin ratio (FPR), prognostic nutritional index (PNI) and albumin-to-alkaline phosphatase ratio (AAPR) (3–7).

Fibrinogen is a glycoprotein synthesized by liver cells and belongs to the acute phase positive protein (8). Fibrinogen levels are elevated during infection or inflammation and play an important role in clotting, cell attachment and thrombosis (9). During the tumor-associated inflammatory response, many events that promote tumor growth and metastasis often occur, including increased release of cytokines and inflammatory mediators, inhibition of apoptosis, and the exertion of immunosuppressive effects (10). Plasma fibrinogen levels have been reported to be associated with tumor progression and prognosis, such as colorectal, endometrial, hepatocellular and pancreatic cancers (11–14). Albumin is produced by the liver and is considered a negative acute phase protein (15). Plasma albumin plays an important role in regulating plasma osmotic pressure, antioxidant, capillary permeability and immune regulation (16, 17). A growing number of studies have shown an association between plasma albumin concentrations, inflammation, and tumorigenesis (18, 19). Available evidence suggests that hypoproteinemia is strongly associated with poor quality of life in cancer patients and is associated with poor prognosis in patients with various malignancies (20, 21). High fibrinogen and low albumin are associated with poor prognosis in cancer patients (12, 22). As a simple and effective new independent predictor, the fibrinogen-to-albumin ratio (FAR) has more prognostic value than high fibrinogen or low serum albumin. FAR has been reported as a potential predictor of adverse outcomes in various malignancies, such as esophageal squamous cell carcinoma, hepatocellular carcinoma, and non-small cell lung cancer (23–25).

A meta-analysis describing the relationship between fibrinogen and albumin ratio and cancer prognosis, exploring the effect of fibrinogen to albumin ratio (FAR) combined with albumin to fibrinogen (AFR) on cancer OS and DFS (26). However, this meta-analysis did not independently explore the effect of fibrinogen-to-albumin ratio (FAR) on cancer prognosis. Therefore, this study included 19 studies for meta-analysis, and evaluated the prognostic significance of FAR in human malignant tumors for the first time.

## Materials and methods

### Search strategy

In order to investigate the potential role of FAR in the prognosis of malignant tumors, this study followed the preferred reporting program of the systematic review and meta-analysis (PRISMA) guidelines (27) and searched the Cochrane Library, Embase, PubMed, Google Scholar, baidu scholar, CNKI and VIP databases. Search for relevant studies published no later than April 1, 2022, regardless of language. Combining the main keywords and free words, the complete search strategy is as follows: (“fibrinogen-to-albumin” OR “fibrinogen/albumin” OR “fibrinogen” OR “albumin”OR “FAR”) AND (“neoplasms” OR “carcinoma” OR “cancer” OR “tumor”) AND (“survival” or “recurrence” or “prognosis” or “progress”). In addition, the references of the retrieved publications are reviewed again to explore more potential relevant research.

### Inclusion and exclusion criteria

The inclusion criteria are shown below:(1)the paper reports the relationship between fibrinogen-to-albumin ratio and long-term prognosis of patients with malignant tumor; (2)prognostic endpoints included overall survival (OS), progression-free survival (PFS), disease-free survival (DFS), recurrence-free survival (RFS) or time to recurrence (TTR);(3) hazard ratio (HR) and 95% confidence interval (CI) are provided or we can calculate it by Kaplan-Meier curve;(4) study patients were divided into two groups based on FAR. Exclusion criteria are listed:(1) hazard ratio (HR) and 95% confidence interval (CI) are not included; (2) outcomes—studies without primary or secondary results; (3) different articles published on the same cohort of patient data; (4) expert opinions, abstracts, case studies, letters or reviews.

### Data extraction and quality assessment

Two researchers (Baibei Li and Huachu Deng) used standardized forms to extract relevant data from the study independently, and if there was a disagreement, all the authors negotiated and resolved it. The basic information extracted included first author name, publication year, country, study type, tumor type, sample size, tumor stage, age, sex, treatment, cut-off, follow-up date, outcome indicators, hazard ratio (HR) and 95% confidence interval (CI). The researcher actively contacted the original author of the included literature to confirm the accuracy of the data and the process of data extraction by the original author. Each included study was scored according to the Newcastle-Ottawa Scale (NOS) (28) criteria to assess its quality. The total score of NOS ranged from 0 to 9, including patient selection (0-4), comparability (0-2) and outcome (0-3). If the NOS score was higher than 6, it was considered to be of high quality in methodology.

### Statistical analysis

STATA (version 12) was used for data analysis, and the combined hazard ratio (HR) and 95% confidence interval (CI) were calculated. Cochran’s Q test and Higgins I^2^ test were used to evaluate the heterogeneity of each study. When I^2^ > 50% or P < 0.10, there was significant heterogeneity, so the random effect model was used. Otherwise, the fixed effect model is adopted. Subgroup analysis was used to evaluate the sources of heterogeneity, and sensitivity analysis was used to determine the reliability and stability of the results. Begg’s test and Egger’s were used to test whether there was publication bias. When P > 0.05, there was no publication bias, otherwise trim-and-fill method would be used for re-evaluation. In this study, all the tests were bilateral, and P < 0.05 was considered to be statistically significant.

## Results

### Study characteristics

**Figure 1:**
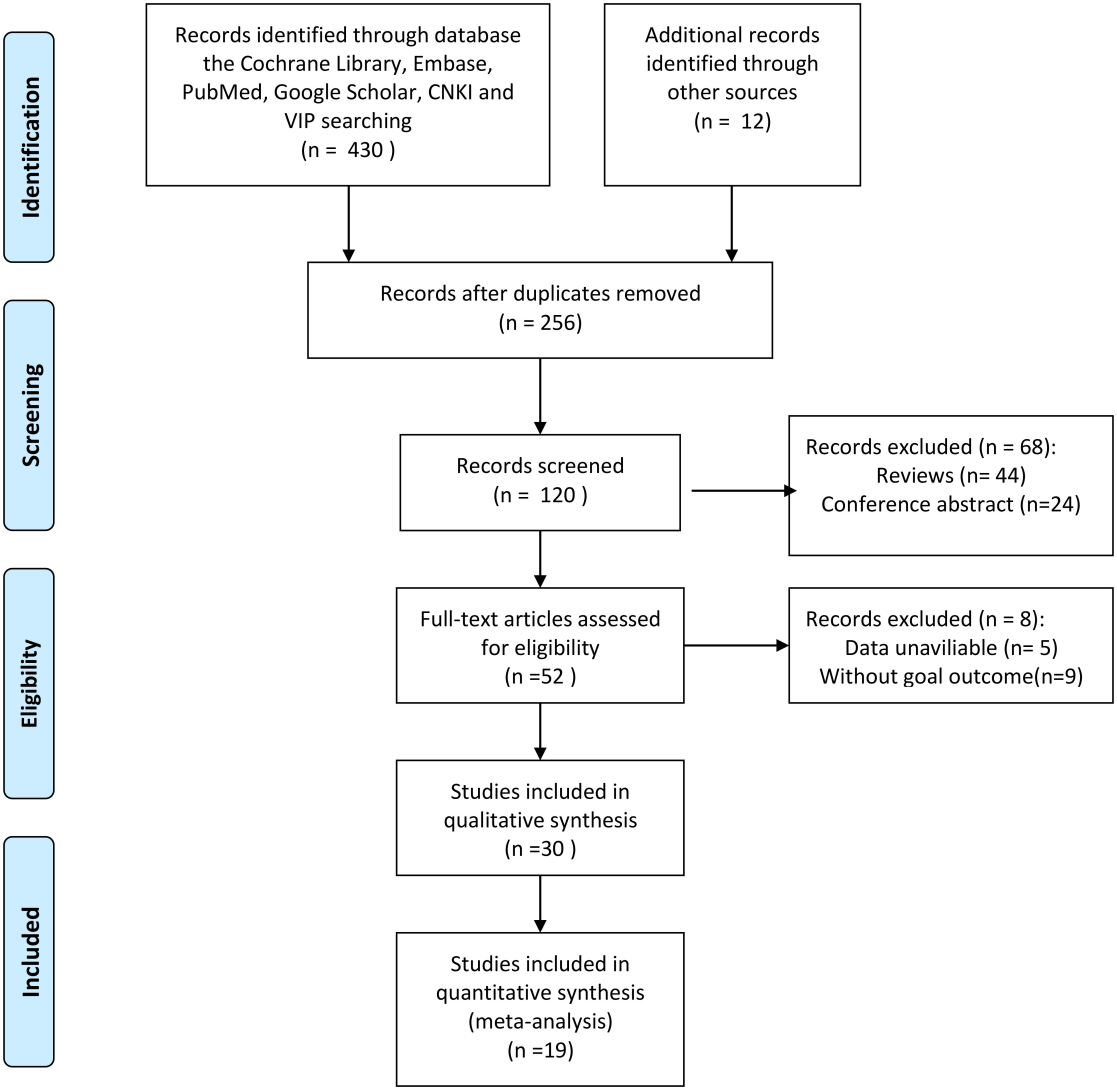
Flow diagram of study collection.

**Figure 1** shows the screening process of the literature included in this study. In the aggregate, a total of 430 articles were retrieved through search strategies, and 12 articles were retrieved through additional records identified through other sources. After duplicates were removed, 256 articles remained. After reading the literature titles and abstracts, 44 reviews and 24 conference abstracts were deleted. Afterwards, we evaluated the full text of the articles and found that the data of 5 of them was of no value, and 9 of the articles did not mention the target result. Therefore, 19 studies involving 5926 cases were included in our meta-analysis (23–25, 29–44). Of the included studies, 18 were from China and one was from South Korea. Publication years are from 2017 to 2022. Sample sizes ranged from 91 to 1,135. In addition, 15 cohorts reported OS, 5 cohorts reported RFS, 4 cohorts reported PFS, 2 cohorts reported DFS, and 1 cohort reported TTR. The baseline information is shown in **Table 1**. The quality of each included article was evaluated by NOS, and the results showed that the scores of these studies were all≥6, indicating that the quality of the included study was high (**Table 2**).

**Table 1:** The characteristics of included studies.

Study	Year	Country	Cancer site	Sample	Gender ratio	Treatment	Outcome	Optimal cut-off for FAR	Follow-up(months)
Zihui Tan et al	2017	China	ESCC	1135	474 /151	Surgical	OS	0.08 by X-tile	More than 60
Kitae Hwang et al	2017	Korea	BC	793	54.1 ± 12.3	Surgical	OS	0.071 by ROC	More than 60
Qiaodong Xu et al	2018	China	HCC	151	128/ 23	Surgical	OS, TTR	0.062 by ROC	More than 60
Weiyu Xu et al	2018	China	Gbc	154	63/91	Surgical	OS	0.08 by ROC	More than 12
Yao Liang et al	2018	China	STS	310	174/135	Surgical	OS	0.0726 by ROC	More than 60
Yanyan Wang et al	2019	China	CRLM	452	289/163	Surgical	OS,DFS	0.076 by X-tile	More than 60
Jun Liu et al	2020	China	RCC	279	195/84	Surgical	OS	0.116 by ROC	More than 60
Lipeng Zhang et al	2020	China	PDAC	282	151/131	Surgical	OS	0.08 by ROC	More than 60
Qiang An et al	2020	China	CC	278	45.5 ± 6.3	mixed	OS,RFS	0.0775 by ROC	More than 60
Siyi Lu et al	2020	China	LARC	123	88/35	mixed	DFS	0.088 by ROC	More than 60
Rui Li et al	2020	China	GIST	227	124/103	Surgical	RFS	0.09 by ROC	More than 60
Xianglong Cao et al	2020	China	GISTs	357	60.88 ±12.05	Surgical	RFS	0.08 by ROC	More than 60
Haitao Yu et al	2021	China	ICC	116	51/65	Surgical	OS, RFS	0.0875 by X-tile	More than 60
Hu Liu et al	2021	China	ICC	394	191/203	Surgical	RFS	0.084 by ROC	More than 36
Shengming Deng et al	2021	China	Pan-NENs	324	142/182	mixed	OS,PFS	0.08 by ROC	More than 36
Jiangang Chen et al	2021	China	BCa	140	120/20	Surgical	OS,PFS	0.08 by X-tile	More than 36
Junhong Li et al	2021	China	GBM	206	133/73	Surgical	OS	0.068 by ROC	More than 12
Chengliang Yuan et al	2022	China	NSCLC	91	68/23	mixed	OS,PFS	0.145 by ROC	More than 24
Wei Chen et al	2022	China	OCCC	114	NA	Surgical	OS,PFS	0.12 by ROC	More than 60

ESCC, esophageal squamous cell carcinoma; BC, breast cancer; HCC, hepatoma carcinoma cell; Gbc, gallbladder cancer; STS, soft tissue sarcoma; CRLM, colorectal liver metastases; RCC, renal cell carcinoma; PDAC, pancreatic ductal adenocarcinoma; CC, cervical cancer; LARC, locally advanced rectal cancer; GIST, gastrointestinal stromal tumor; ICC, intrahepatic cholangiocarcinoma; Pan-NENs, pancreatic neuroendocrine neoplasms; BCa, bladder cancer; GBM, glioblastoma; NSCLC, non-small cell lung cancer; OCCC, ovarian clear cell carcinoma; NA, not available.

**Table 2:** Newcastle–Ottawa scale for quality assessment.

Study	Selection				ComparabilityControl for factor		Outcome		Total score
	Exposedcohort	Non-exposedcohort	Ascertainment of exposure	Outcomeof interest		Assessmentof outcome	Follow-uplong enough	Adequacyof follow-up	
Zihui Tan	*	*	*	*	*	*	*		7
Kitae Hwang	*	*	*	*	*	*	*	*	8
Qiaodong Xu	*	*	*	*	*	*	*	*	8
Weiyu Xu	*	*	*	*	**	*		*	8
Yao Liang	*	*	*	*	*	*	*	*	8
Yanyan Wang	*	*	*	*	**	*	*		8
Jun Liu	*	*	*	*	**	*	*		8
Lipeng Zhang	*	*	*	*	*	*	*	*	8
Qiang An	*	*	*	*	**	*			7
Siyi Lu	*	*	*	*	*	*	*		7
Rui Li	*	*	*		**	*		*	7
Xianglong Cao	*	*	*	*	*	*	*	*	8
Haitao Yu	*	*	*		**	*		*	7
Hu Liu	*	*	*	*	**	*	*	*	9
Shengming Deng	*	*	*	*	**	*	*	*	9
Jiangang Chen	*	*	*		*	*	*		6
Junhong Li	*	*	*	*	**	*	*		8
Chengliang Yuan	*	*	*	*	**		*	*	8
Wei Chen	*	*	*	*	**		*		7

Maximum amount of stars for Selection is 4; Maximum amount of stars for Comparability is 2; Maximum amount of stars for Outcome is 3; Maximum amount of stars for Total Score is 9. A Total score of 0 –3 indicates high risk, 4 –6 a moderate risk, and 7 –9 a low risk of bias. Each "* represents a point.

### FAR and overall survival

**Figure 2:**
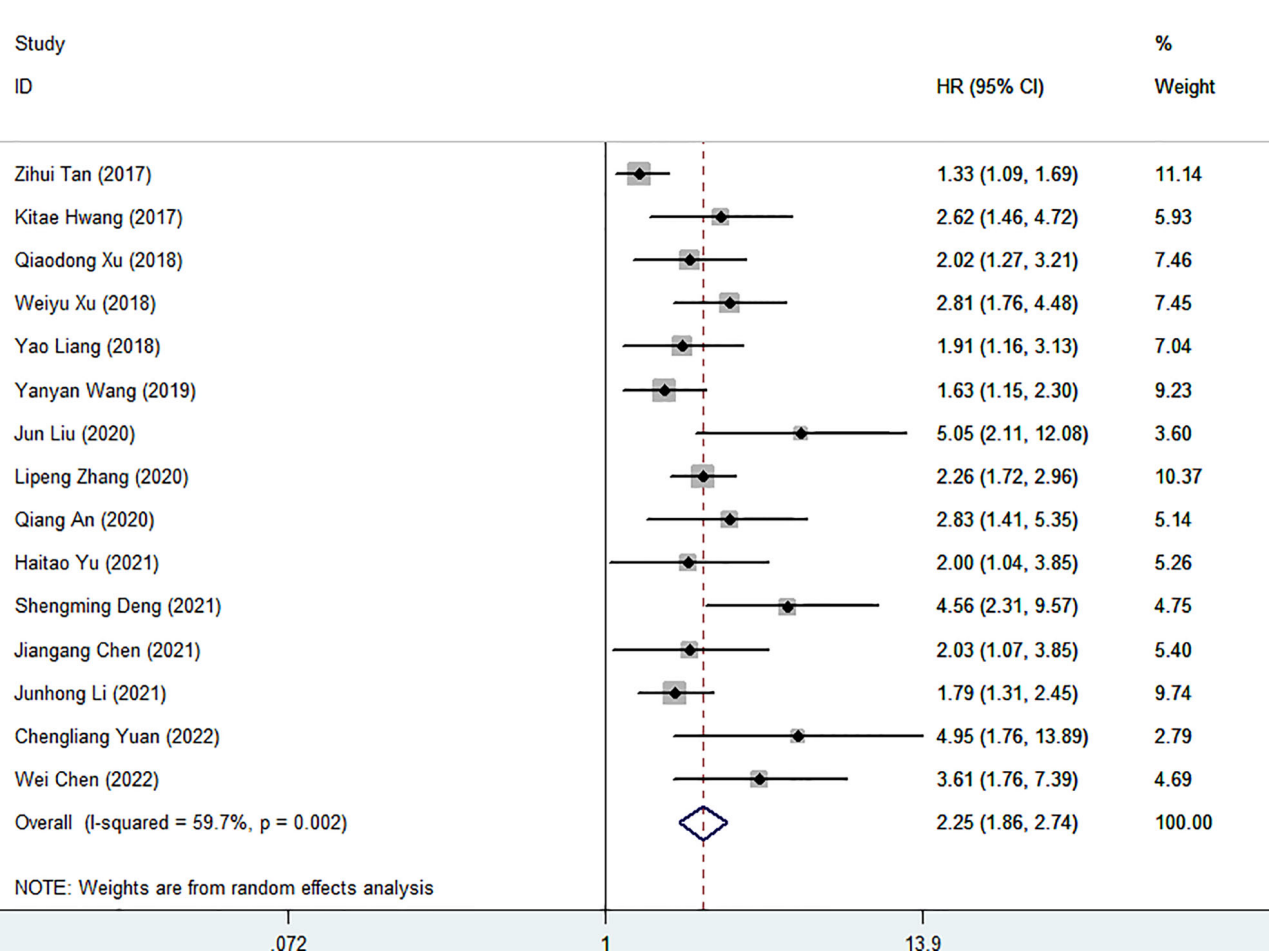
Forest plot for the association between FAR and overall survival.

Fifteen cohort studies involving a total of 4825 patients revealed the prognostic effect of FAR levels on human malignant tumor OS. Our results show that the relatively high level of HRR before treatment is related to the decrease of OS (HR=2.25, 95%CI 1.85-2.74, p<0.001). Because of the significant heterogeneity, the random effect model is adopted (I^2 =^ 59.7%, P=0.002) (**Figure 2**). Therefore, we performed a stratified subgroup analysis by cancer type, treatment option, sample capacity, publishing time, methods for choosing FPR cut-off value and cut-off value to explore the source of heterogeneity (**Table 3**). Despite the differences between groups, high FAR was significantly associated with poor OS. Furthermore, some subgroup heterogeneity was eliminated when we stratified according to factors such as “Gynecology”, “Mixed”, “Cut-off value > 0.08”, “Sample capacity ≤ 250” and “X-tile”. Further analysis of these results led us to suggest that the use of mixed treatment option and the large sample capacity may have contributed to the heterogeneity.

**Table 3:** Subgroup meta-analysis of FAR and OS.

					Heterogeneity	
Subgroup	No.of cohorts	No. of patients	Pooled HR (95% CI)	P	*I* ^2^ (%)	P_h_
Altogether	15	4825	2.25 (1.86,2.74)	0.000	59.7	0.002
Cancer types						
Digestive	7	2614	2.06 (1.56,2.71)	0.000	71.1	0.002
Urinary	2	419	3.04 (1.25,7.39)	0.014	63.2	0.099
Gynecology	2	392	3.17 (1.94,5.16)	0.000	0.0	0.628
Others	4	1400	2.04 (1.61,2.57)	0.000	29.5	0.235
Treatment option						
Surgical	12	4132	2.06 (1.71,2.49)	0.000	55.0	0.011
Mixed	3	693	3.76 (2.42,5.84)	0.000	0.0	0.535
Sample capacity						
≤250	7	972	2.19 (1.81,2.66)	0.000	16.4	0.304
>250	8	3853	2.25 (1.67,3.02)	0.000	72.6	0.001
Publishing time						
≤2020	9	3834	2.12 (1.67,2.68)	0.000	64.5	0.004
>2020	6	991	2.60 (1.82,3.73)	0.000	49.6	0.077
Methods for choosing FPR cut-off value						
X-tile	4	1843	1.48 (1.25,1.76)	0.000	0.7	0.388
ROC	11	2982	2.38 (2.06,2.74)	0.000	31.9	0.144
Cut-off value						
≤0.08	11	4225	2.06 (1.70,2.50)	0.000	57.9	0.008
>0.08	4	600	3.27 (2.21,4.84)	0.000	21.2	0.283

### Sensitivity analysis and publication bias for OS

**Figure 3:**
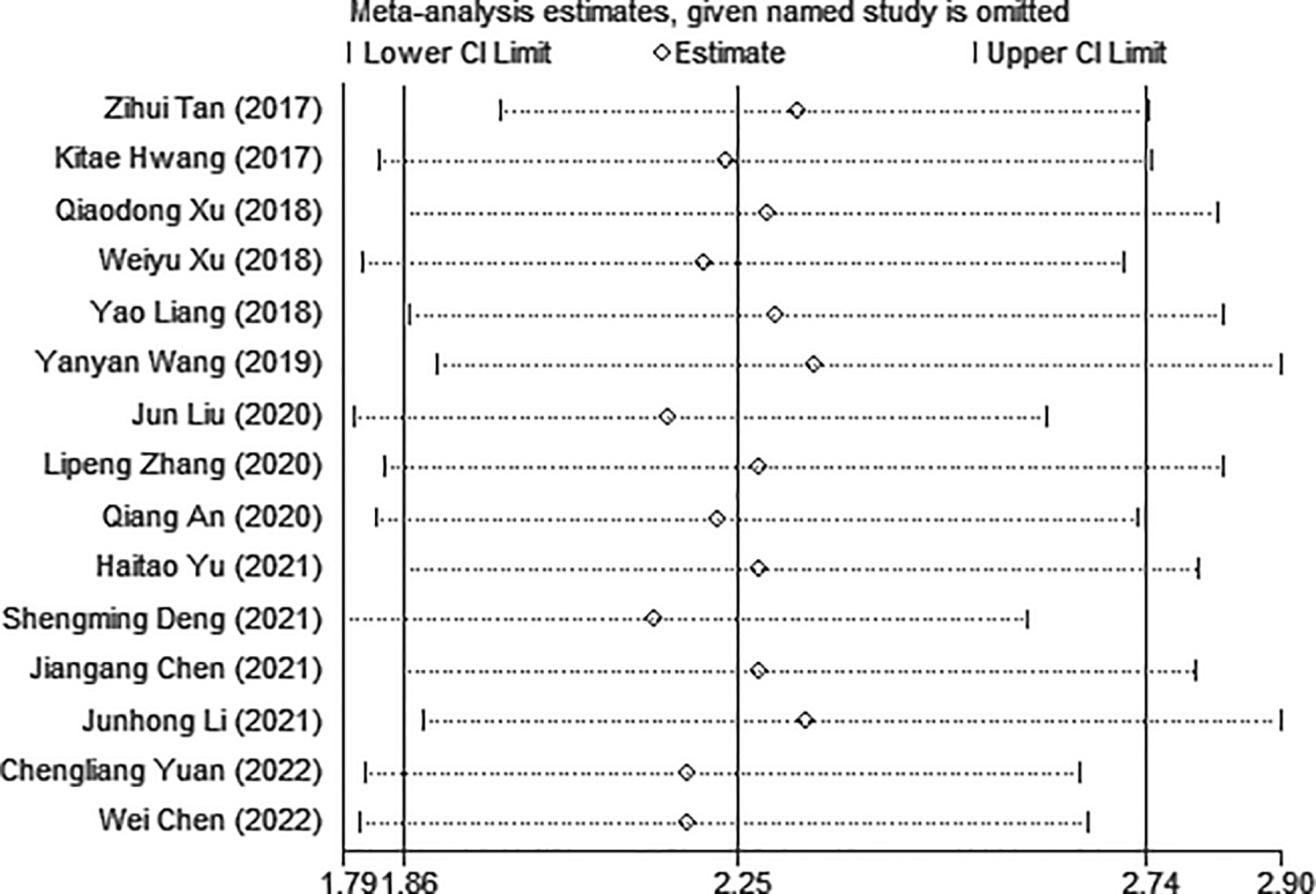
Sensitivity analysis for the association between FAR and OS. OS, overall survival.

**Figure 4:**
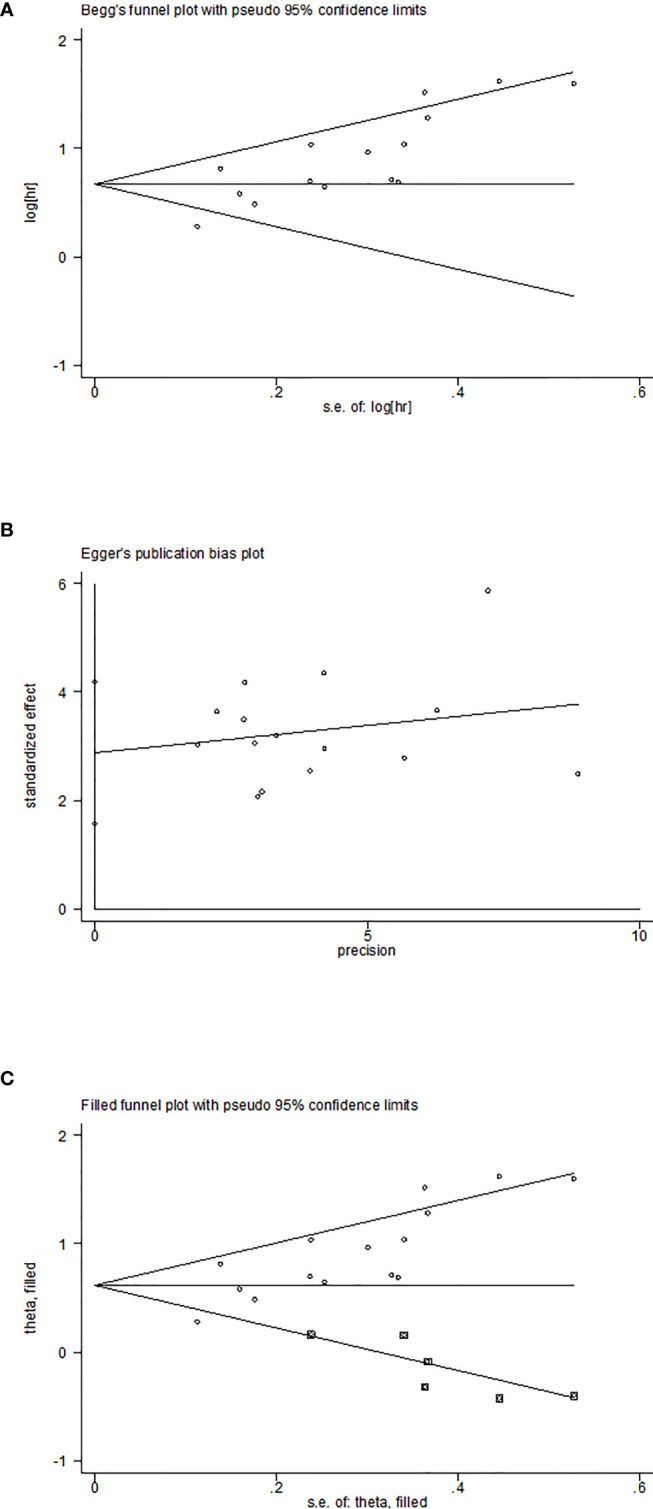
Plots for publication bias test in meta-analysis for overall survival. **(A)**Begg’s funnel plot; **(B)** Egger’s publication bias plot; **(C)** The trim-and-fill methods.

Sensitivity analysis was used to explore the potential impact of each study on the combined results. After we removed each study separately, we recalculated the combined HR and its 95%CI.The results show that ignoring any study will not significantly change the effect of FAR on OS joint meta-analysis, in other words, the comprehensive results of our meta-analysis are stable (**Figure 3**). In the meta-analysis of OS, Begg’s test (p = 0.006) and Egger’s test (p = 0.000) suggested potential publication bias. Therefore, the trim-and-fill method is further used for correction. The symmetrical funnel diagram is obtained by adding six studies, and the corrected HR is still significant (HR=1.853, 95%CI 1.522-2.257, p<0.001), indicating that our results are reliable (**Figures 4A–C**).

### FAR and recurrence−free survival

**Figure 5:**
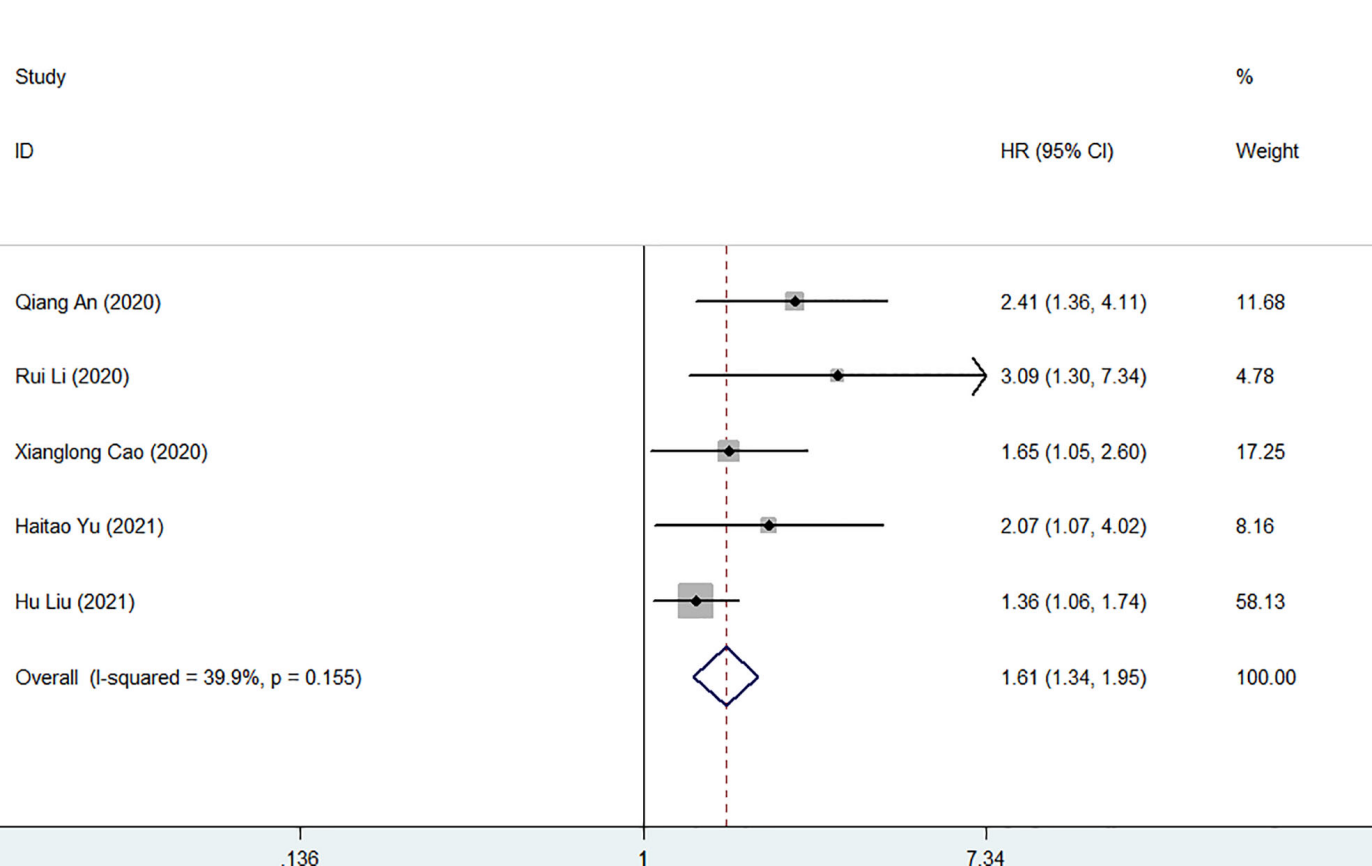
Forest plot for the association between FAR and recurrence−free survival.

Five studies involving 1372 patients reported the association between FAR and postoperative recurrence-free survival in patients with malignancy. The comprehensive results showed that a high FAR was related to poor RFS in patients with malignant (HR = 1.61, 95% CI 1.34-1.95, p < 0.001). In the case of low heterogeneity (I^2^ = 39.9%, P = 0.155), we used a fixed-effect model (**Figures 5**). Furthermore, we performed subgroup analysis according to cancer type, sample capacity, publishing time, sample capacity, methods for choosing FPR cut-off value, sample capacity, cut-off value and treatment option. The results showed that FAR was an independent prognostic factor affecting RFS in each subgroup (**Table 4**).

**Table 4:** Subgroup meta-analysis of FAR and RFS.

					Heterogeneity	
Subgroup	No.of cohorts	No. of patients	Pooled HR (95% CI)	P	*I* ^2^ (%)	P_h_
Altogether	5	1372	1.61 (1.34,1.95)	<0.001	39.9	0.155
Cancer types						
ICC	2	510	1.43 (1.13,1.80)	0.003	27.6	0.24
GIST	2	584	1.89 (1.26,2.83)	0.002	37.1	0.207
CC	1	278	2.41 (1.36, 4.11)	0.002	NA	NA
Sample capacity						
≤250	2	343	2.40 (1.42,4.06)	0.001	0.0	0.470
>250	3	1029	1.63 (1.19,2.22)	0.002	44.7	0.164
Publishing time						
≤2020	3	862	2.06 (1.49,2.85)	<0.001	3.5	0.355
>2020	2	510	1.43 (1.13,1.80)	0.003	27.6	0.240
Methods for choosing FPR cut-off value						
ROC	4	1256	1.78 (1.27,2.49)	0.001	50.6	0.108
X-tile	1	116	2.07 (1.07,4.02)	0.031	NA	NA
Cut-off value						
≤0.08	2	635	1.92 (1.35,2.73)	<0.001	7.0	0.300
>0.08	3	737	1.80 (1.12,2.87)	0.015	52.9	0.120
Treatment option						
surgical	4	1094	1.53 (1.25,1.87)	<0.001	31.5	0.223
mixed	1	278	2.41 (1.36,4.11)	0.002	NA	NA

### Association between FAR and other outcomes

**Figure 6:**
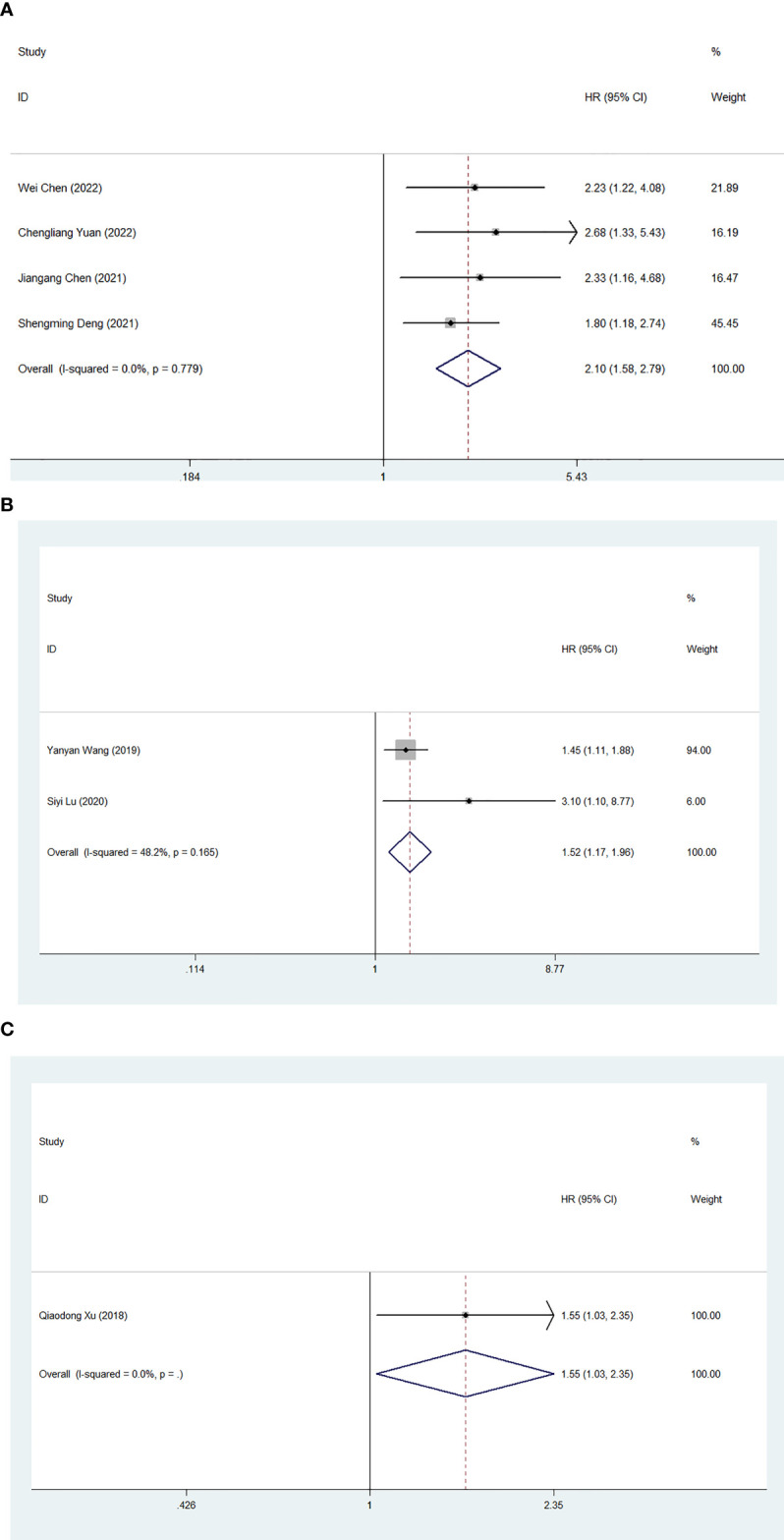
Forest plot for the association between FAR and progression-free survival **(A)**/disease-free survival **(B)**/time to recurrence **(C)**.

We also investigated the effect of FAR on PFS, DFS and TTR in human patients with malignant tumors. Four studies involving 669 medical records reported the prognostic impact of FAR on PFS. Due to the lack of heterogeneity, a fixed-effects model was used (I^2 =^ 0.0%, P=0.779). Higher FAR was a prognostic factor for poor PFS in patients with human malignancies (HR: 2.10, 95% CI 1.58-2.79, p<0.001) (**Figure 6A**). Two studies involving a total of 575 patients reported the relationship between FAR and DFS in patients with malignancy, and the combined results showed that high FAR was an independent risk factor for DFS in patients with malignancy (HR=1.52, 95%CI 1.17-1.96, p= 0.001) (**Figure 6B**). Additionally, a study involving 151 medical records reported the prognostic effect of FAR on TTR, and FAR was also a prognostic factor for poor TTR in patients with human malignancies (HR: 1.555, 95% CI 1.031-2.346, P=0.035) (**Figure 6C**).

## Discussion

In recent years, there have been continuous studies to explore the relationship between FAR and the clinical outcome of human solid tumors (29, 35, 36, 39). FAR is expected to be a simple, inexpensive, and readily available biomarker for predicting clinical prognosis in patients with solid tumors. However, the underlying mechanisms of how FAR influences cancer prognosis remain unclear. As a composite indicator based on fibrinogen and albumin, FAR can explain its mechanism of action in cancer prognosis by studying the functions of its components.

When the body is under pathophysiological states such as tumor, surgery, infection, inflammation, trauma, etc., the level of fibrinogen increases to varying degrees (45). Studies have shown that fibrinogen plays a cytoskeleton role in tumor extracellular matrix, protecting tumor cells from being killed by immune cells (46, 47). Jay S Desgrosellier et al. showed that fibrinogen can act as a bridge between platelets and circulating tumor cells (CTCs), promote platelet adhesion to CTCs, and increase the metastatic potential of tumor cells (48). In addition, fibrinogen can also directly bind to the intercellular adhesion molecule-1 (ICAM-1) of endothelial cells to promote tumor cell adhesion, proliferation and migration (49). Many studies have shown that tumor cells can synthesize and secrete additional endogenous fibrinogen, and high fibrinogen promotes the synthesis of IL-6, thereby stimulating T and B cells to promote chronic validation responses (50, 51). In animal experiments, tumor cell metastasis and migration were significantly inhibited in fibrinogen-deficient mice (52).

Previous studies have shown that the nutritional status of cancer patients is related to the patient’s age, degree of disease progression and prognosis (53). Albumin, the most abundant circulating protein in plasma, not only reflects the nutritional status of the human body, but also participates in systemic inflammatory responses (54). Low albumin levels may lead to impaired immune function in tumor patients and promote tumor proliferation, invasion and migration (55). Studies have shown that albumin deficiency is closely related to postoperative complications, secondary operations and recurrence of malignant tumors (56). In addition, Christopher G Lis et al. reported that albumin may help stabilize DNA replication and cell growth, regulate body responses, enhance natural immunity, and prevent malignant diseases (57).

Many biomarkers have been shown to be associated with OS, PFS, etc. in cancer, including neutrophil-to-albumin ratio (NAR), neutrophil-to-lymphocyte ratio (NLR), and C-reactive protein-to-albumin ratio (58–60). Available evidence suggests that fibrinogen and albumin are considered independent prognostic indicators for human solid tumors (61, 62). The FAR value derived from the ratio of these two indicators may combine the predictive effects of these indicators, reflecting a mixed prognostic value (40). Therefore, FAR is not only related to systemic inflammation, but also to coagulation and nutritional status (23). A study of 273 patients with advanced gastric cancer showed that FAR was considered a valuable predictor of PFS and OS, and was superior to fibrinogen or albumin alone (63). Junhong Li et al. reported that FAR was better than fibrinogen and albumin in predicting the prognosis of glioblastoma patients (30).

Here, we included 19 studies involving 5926 patients with malignancies. Available evidence suggests that FAR is a sensitive predictor of prognosis in human malignancies, with patients with high FAR exhibiting worse OS than patients with low FPR. At the same time, we also performed subgroup analyses to explore the influence of various factors on our final conclusions. Despite clear differences between subgroups, high FAR was still significantly associated with poor prognosis. The stability of the meta-analysis was further verified by sensitivity analysis and publication bias. In addition, we further discuss the relationship between FAR and RFS. The combined results showed that FPR was an independent predictor of RFS in patients with malignancy. Meanwhile, subgroup analysis showed that despite differences in different subgroups such as cancer types, sample capacity, publishing time, sample capacity, methods for choosing FPR cut-off value, sample capacity, cut-off value and treatment option, high FAR still significantly associated with poorer RFS. In addition, we discuss the relationship of FPR to other prognostic indicators of malignancy, with higher FPR being associated with poor clinical outcomes in PFS, DFS and TTR. In conclusion, FAR can be considered as an important and practical clinical indicator for predicting the prognosis of patients with malignant tumors. In addition, the critical value of FAR in most of the included studies was 0.08, which provided a certain reference value for the application of FAR in clinical work.

However, the results of our meta-analysis should be interpreted with caution, considering some limitations. First, despite the use of subgroup analyses and sensitivity analyses, sources of heterogeneity could not be fully traced. Second, all the included studies were conducted in Asia, which may affect its applicability in other populations when applied on a large scale. More studies from other regions are needed in the future to confirm its applicability in the whole human race. Third, only aggregated data were included, and data from individual patients were not provided for analysis. Fourth, including only English-language studies may introduce some bias. Despite these limitations, based on the available evidence, we have meaningfully explored the prognostic value of preconditioning FAR in cancer patients.

## Conclusion

In summary, the outcomes of this meta-analysis shed light on that a higher level of pre-treatment FAR was positively associated with OS, RFS, PFS, DFS and TTR, indicating that it could be an independent prognostic factor in human solid tumors.

### Future perspectives

Due to limited research, it restricted our in-depth investigation of the role of FAR. Hence, larger samples with higher quality randomized controlled trials were required to verify our findings. FAR consists of fibrinogen and albumin, both of which are commonly used laboratory tests that are easy and inexpensive to obtain. In the future, if FAR can be used in clinical treatment, it will improve the efficiency of diagnosis and reduce the cost of treatment for cancer patients.

## Data availability statement

The original contributions presented in the study are included in the article/supplementary material. Further inquiries can be directed to the corresponding author.

## Author contributions

BBL and HCD contributed equally to this work. BBL and HCD designed this research. BL and LJC performed the statistical analysis. XYZ and DRS performed the data extraction, and drafted and revised the manuscript. All authors contributed to the article and approved the submitted version.

## Acknowledgments

We would like to thank the researchers and study participants for their contributions.

## Conflict of interest

The datasets generated during and/or analyzed during the current study are available from the corresponding author on reasonable request.

## Publisher's note

All claims expressed in this article are solely those of the authors and do not necessarily represent those of their affiliated organizations, or those of the publisher, the editors and the reviewers. Any product that may be evaluated in this article, or claim that may be made by its manufacturer, is not guaranteed or endorsed by the publisher.
